# Latvian Healthcare Professionals’ Self-Reported Knowledge, Attitudes, and Behaviors Related to Pregnancy Prevention Program Materials for Valproate-Containing Medicines

**DOI:** 10.3390/pharmacy12060182

**Published:** 2024-12-04

**Authors:** Milana Špoģe, Mirdza Kursīte, Elita Poplavska

**Affiliations:** 1 Department of Applied Pharmacy, Faculty of Pharmacy, Riga Stradiņš University, LV-1007 Riga, Latvia; 2 Institute of Public Health, Riga Stradiņš University, LV-1010 Riga, Latvia; mirdza.kursite@rsu.lv; 3 Department of Applied Pharmacy, Faculty of Pharmacy, LV-1007 Riga, Latvia and Institute of Public Health, Riga Stradiņš University, LV-1010 Riga, Latvia; elita.poplavska@rsu.lv

**Keywords:** valproates, Pregnancy Prevention Program, healthcare professionals, survey

## Abstract

Background: Valproates are recognized for their significant teratogenic risks, which can lead to physical defects and developmental disorders when used during pregnancy. To mitigate these risks, the Pregnancy Prevention Program (PPP) was developed by European regulators for patients and healthcare professionals (HCPs). Despite the crucial nature of this program, the implementation of the PPP does not appear to be fully effective. This situation highlights the need for a deeper understanding of HCPs’ knowledge, attitudes, and behaviors regarding the PPP. Methods: A cross-sectional study using anonymous electronic questionnaires was conducted. The questionnaires were developed by a board of experts from eight different EU countries and were distributed among prescribers (general practitioners (GPs), neurologists, and psychiatrists) and pharmacists. Descriptive statistics were used to analyze the obtained data on participants’ knowledge, attitudes, and behaviors regarding the prescribing and dispensing of valproate-containing medicines to women of reproductive age, as well as the impact of PPP materials on their work. Results: The study results indicate that while HCPs in Latvia are generally aware of valproate teratogenic risks, significant gaps remain in the implementation of the PPP. A considerable number of both prescribers and pharmacists expressed the belief that they are not responsible for educating patients about the PPP, attributing this responsibility to other specialists. Furthermore, barriers such as a lack of time and accessible materials were identified. Conclusions: The roles and responsibilities of HCPs should be clearly defined to improve adherence to the PPP. Further research is needed to assess prescription and dispensing strategies, as well as HCPs’ attitudes toward the PPP. Additionally, re-evaluating and enhancing the accessibility of PPP materials is essential in effective risk management and better patient care.

## 1. Introduction

Certain medications stand out due to their potential for harm, given their specific characteristics and possible side effects, highlighting the need for special attention. These medications include valproates and related substances. The teratogenic effects of valproates are well established and can lead to physical defects and developmental disorders when used during pregnancy [1]. To prevent such risks, the Pregnancy Prevention Program (PPP) was developed by European regulators for both patients and healthcare professionals. The main aims of the PPP regarding valproate use are (1) to inform medication users about the possible risks and their mitigation options and (2) to ensure that prescribers and community pharmacists are aware of the teratogenic risks when prescribing or dispensing these drugs [2]. The PPP for valproate-containing medicines consists of three parts: an educational program, the evaluation and control of therapy, and medication distribution control. For the educational program, a set of documents for HCPs—designed differently for prescribers and pharmacists—were developed in agreement with the competent authorities of the European Medicines Agency (EMA) and the EU member states [2]. Each member state adapted the materials according to national needs.

Latvia is a country in the European Union with a population of approximately 1.9 million people. There are 2.3 female users of valproates and valproate-containing medicines per 1000 females in Latvia, which is similar to other countries in the region [3]. Within the framework of the national drug reimbursement system, these medicines are fully reimbursed for the treatment of epilepsy, while for the treatment of bipolar disorders and various off-label psychiatric indications, a 25% co-payment is applicable [4]. A list of PPP materials approved in Latvia is presented in Table 1.

The PPP includes the provision of educational information (both in paper form and verbally) for patients, the performance of regular pregnancy tests under medical supervision, and the prescription of at least one effective contraceptive method (preferably two, with one of the methods being a barrier method) before the start of therapy, during the entire therapy, and at least 4–5 weeks after the end of therapy [1].

Latvia has implemented additional distribution control measures whereby valproate-containing medicines can be prescribed only through special prescription forms, and the prescription is valid for 90 days [5].

Despite the critical importance of PPP, it can still be quite challenging for HCPs to fully implement it into their practice [6,7,8,9]. Evidence from studies examining the PPP for oral retinoids highlights that, despite having similar requirements, the implementation of the program has also been insufficient and requires substantial improvements [6,7,8,10,11,12]. Since the introduction of the PPP for valproates in 2018, several studies on PPP have attempted to measure the PPP’s impact, and the results of these studies demonstrate that a noticeable number of women still become pregnant while taking valproates despite a high level of awareness about teratogenic risks among HCPs [9,13,14,15].

Effective risk communication regarding valproate-containing medicines is crucial, as insufficient understanding of associated risks can lead to severe consequences. Existing guidelines should be followed rigorously to minimize risks, particularly from HCPs’ side, as they are typically better educated and informed on this topic compared to patients. Overall, there is a rather limited number of studies specifically examining the roles and perspectives of HCPs in the process of PPP implementation. Therefore, it is important to examine HCPs’ knowledge, attitudes, and behaviors in relation to the current valproate PPP in order to evaluate overall adherence to the PPP requirements and understand how these could be improved. The aforementioned aspects were studied in a multicountry survey among eight EU countries (Belgium, Denmark, Greece, Latvia, Portugal, The Netherlands, Slovenia, and Spain) as part of a larger study [16]. This paper presents the results obtained for Latvian HCPs based on the survey findings.

## 2. Materials and Methods

### 2.1. Questionnaire Development

Two questionnaires, adapted for the corresponding respondent groups (pharmacists vs. prescribers), were developed by a board of experts from eight EU countries—Belgium, Denmark, Greece, the Netherlands, Portugal, Slovakia, Spain, and Latvia—as part of the international post-registration safety analytical study. The questionnaires were developed in English with the participation of Latvian experts and were then translated into Latvian. They were piloted before distribution. The questionnaires were placed on the webpage of the online survey tool LimeSurvey, which respondents could access by clicking a link that they were sent.

HCPs’ knowledge, attitudes, and behaviors regarding valproate-containing medications, as well as the impact of PPP materials on their practice, were assessed using a total of 18 questions. The questions were thematically organized into groups: demographic information (questions 1–5); knowledge of contraindications for the use of these drugs during pregnancy and related PPP materials (questions 6–9); habits regarding the use of PPP materials and prospects for future use (questions 10–12); and practices for providing patient information (questions 13–16). The responses to questions about knowledge and attitudes comprised self-reported evaluations using a Likert scale. Questions 17 and 18 were optional open-ended questions in which respondents were invited to identify obstacles that hinder the implementation of the PPP and to provide recommendations and comments. Each group of questions was placed on a separate page of the survey, and the next page could be opened only after completing the previous one; if anything was missing, it was highlighted in red. Respondents could review their responses by going back to the start of the survey. Additionally, respondents could receive the results of the study by checking the respective box prior to the start of the survey.

### 2.2. Study Population

All participants were recruited from March 2020 to July 2020. Nearly all prescribers of valproate-containing medicines in Latvia and all Latvian chain pharmacies in the country were invited to participate in the questionnaire. A convenience sampling approach was used for the study. Recruitment strategies for pharmacists and prescribers differed slightly due to the specific work characteristics of each participant group. Prescribers were recruited via emails sourced from the National Healthcare System database, utilizing both institutional and private email addresses. Follow-up emails were sent after 4 weeks as reminders to complete the survey. One month after initial outreach, all prescribers listed in the database received telephone calls with follow-up reminders to complete the questionnaire. For pharmacists, information about the study and links to the electronic questionnaires were sent to the email addresses of Latvian pharmacy chains. Follow-up emails were also sent after 4 weeks as reminders to complete the survey. Additionally, information about the study was posted in the largest Latvian pharmacists’ Facebook group, Pharmacists Forum (Farmaceitu forums). Active participants of this group were privately contacted via Facebook and invited to take part in the survey, with a request to share it with other pharmacists, as well. Furthermore, an article containing information about the study and links to the questionnaires was published on the website of Riga Stradiņš University and on the portal of the Medicines Information Center (Medikamentu Informācijas Centrs), an organization that provides informational services to healthcare professionals, such as newsletters and publishing services, in order to reach all medical specialists.

The inclusion criterion for prescribers was the treatment of at least one woman of reproductive age using valproate-containing medications in the past year. For pharmacists, inclusion required employment in a community or open-type hospital pharmacy and experience in dispensing valproate-containing medications. If a respondent did not meet the criteria, then they received a notification stating, “Thank you for your interest; however, you are not part of the population for this study” and were excluded from the survey.

### 2.3. Data Analysis

All responses were saved and stored in the LymeSurvey database. Three main topics were analyzed in relation to the PPP for valproate-containing medications:

- HCP knowledge regarding the PPP (knowledge about the teratogenicity of valproate, information sources, understanding of reproductive age, and awareness of PPP materials);
- HCP attitudes toward the PPP (attitudes regarding the future use of PPP materials, the self-reported impact of PPP materials on their work, and perceived barriers to the implementation of the PPP);
- HCP behaviors related to the PPP (prescribing habits and information provision habits in both prescribers and pharmacists).

Data were analyzed using MS Excel. Descriptive statistical methods were employed to characterize the following parameters: respondents’ demographic information and HCPs’ knowledge, prescribing/dispensing habits, information provision habits, and opinions about the PPP. The categorical variables were reported as absolute and relative frequencies (%). For the responses to the open-ended questions, an inductive content analysis was performed. The responses were examined closely, line by line, and a conceptual coding scheme based on the major themes was developed. The most frequent themes are presented in the Section 3.

### 2.4. Ethical Considerations

Prior to completing the questionnaire, all respondents were informed about the investigators and the goals of the study and the approximate length of the survey and were asked to provide informed consent by ticking the following statement: “I hereby confirm that I have read the above information note, participate voluntarily and give permission to process my answers scientifically”. The respondent could not proceed with the study unless this statement was checked. The electronic survey was completely anonymous, and no personal data were collected, which was also communicated to the respondents prior to the start of the survey. Approval from the ethical committee at Riga Stradiņš University was obtained before the start of the study.

## 3. Results

### 3.1. Demographic Information

In total, 212 prescribers and 55 pharmacists started the questionnaire, of whom 109 prescribers and 7 pharmacists did not meet the inclusion criteria and were excluded from the analysis. Consequently, 103 prescribers and 48 pharmacists were included in the analysis. The median age of prescribers was 52 years, while the median age of pharmacists was 31 years, which is consistent with the results obtained in other EU countries.

Other demographic characteristics, such as their gender, professional category, and duration of practice in their current profession, are summarized in Table 2, below:

### 3.2. Knowledge

The majority of prescribers (55.9%) reported that they learned about the teratogenic effects of valproates more than 5 years ago. The most common information resource for this knowledge was professional organizations or associations (69.4%), except for among neurologists, who most frequently cited symposia or conferences as their primary source of information. Other significant resources included professional journals (45.9%), post-academic training/continuous professional education (42.9%), the State Agency of Medicines (41.8%), and symposia or conferences (40.8%). The internet was identified as the least important information source (Table 3).

On the contrary, pharmacists have only recently become aware of the teratogenicity associated with valproate-containing medications; 47.9% of respondents reported that they had acquired this knowledge within the past two years. The most common information sources for pharmacists were academical training (50.0%) and information from Health Authorities (45.8%), and the least utilized were symposia or conferences (2.08%) (Table 3).

Among all the PPP materials, the discussion and signing of the Risk Acknowledgment Form (RAF)—54.7% and 53.7%, respectively—as well as the Patient Reminder Card (51.6%) were considered the most popular PPP measures for prescribers, while the HCP guide was the least utilized and recognized (Table 4). Although the highest proportion (34.7%) of respondents were familiar with the DHCP letter, they reported that they did not use it. General practitioners (GPs) exhibited the lowest familiarity with PPP materials related to valproate-containing medications among all prescribers; of the 35 GPs who responded, more than half (48.6% for the HCP guide, 54.3% for the patient guide, 57.1% for RAF discussion, and 60.0% for RAF signing) had not encountered any PPP materials except for the patient reminder cards and DHCP letter, with 37.1% and 31.4% indicating that they had not seen even those, respectively.

The warning symbol on the outer package was identified as the most well-known and well-used PPP material among pharmacists (70.8% had used or were using it), while the HCP guide, pharmacist checklist, and patient reminder card were the least recognizable. Almost half of the respondents (45.8%) were familiar with the DHCP letter; however, they did not use it (Table 5).

### 3.3. Attitudes

When asked about intentions to use PPP materials in the future (particularly among those who were previously unfamiliar with the materials or had not used them in the past), prescribers were mostly willing to use them (Table 6). The most favorably perceived measure/material was the signing and discussion of the RAF, with 73.3% and 59.1% reporting their use of them in the future as very likely or likely. The least probably used materials were the healthcare professional guide and DHCP letter, with 29.2% and 14.9%, respectively, indicating that future use was very unlikely or unlikely. Among the reasons why these materials were not likely to be used, the most frequently mentioned factors were a lack of necessity and the unavailability of the materials themselves. Additionally, some prescribers indicated patient attitudes among reasons for not using the materials.

Similar dynamics were also observed among pharmacists regarding the use of PPP materials in the future (Table 7). All respondents indicated that they were most likely to use the warning symbol on the outer carton. The materials that were least likely to be used were the pharmacist checklist, DHCP letter, and patient reminder card. Among the reasons why these materials were not used, the most common were a lack of understanding of what these materials were (the pharmacist checklist), a lack of necessity and time (the DHCP letter), and a lack of availability (the patient reminder card).

In response to the open-ended question about the main obstacles to the use of PPP materials, the following themes were identified among prescribers as the most common: the absence of materials, a lack of time, and the belief that prescribers should not be responsible for educating patients on this topic. Additionally, the lack of convenience of using these materials (e.g., paper materials vs. electronic materials) and the absence of specifically identified obstacles were also mentioned as common themes.

The most commonly cited obstacles to the successful implementation of the PPP, in the opinion of pharmacists, were the lack of materials in the workplace, a lack of time, and a lack of understanding regarding what these materials were.

### 3.4. Behaviors

Overall, specialists positively evaluate the impact of PPP materials in their prescribing practice, with responses indicating “probably yes” and “certainly yes” at rates of 28.57% and 27.47%, respectively. In contrast, almost half of the responding pharmacists (47.3%) admitted that the information that they provide to women of reproductive age about valproate-containing medicines had probably not changed since the introduction of the PPP in 2018 (Table 8).

In response to the open-ended questions regarding what had specifically changed in their practice, two main themes were identified: either specialists chose not to prescribe valproate-containing medicines to patients of reproductive age (or reduced such practices) or they provided more detailed consultations to their patients regarding pregnancy avoidance, often involving gynecologists and other specialists in the process.

The most common reasons for not using materials were the absence of these materials (mentioned for PC and PG) or a lack of necessity (DHCP).

When asked about their current prescribing practices concerning valproate-containing medicines, prescribers indicated that they were selective when prescribing these medications to women of reproductive age; more than half also reported discontinuing therapy if pregnancy was planned or suspected (Table 9). Additionally, 76.8% referred patients to consult a medical specialist if pregnancy was planned or suspected.

Opinions regarding the necessity of monthly follow-up visits for women of reproductive age were divided. No single viewpoint emerged as overwhelmingly favorable, with 28.0% of respondents responding that they rather agreed and 18.3% saying that they rather disagreed. A similar pattern was observed in responses concerning pregnancy testing. While a majority (32.9%) agreed that pregnancy testing was necessary prior to the initiation of treatment, opinions regarding the necessity of monthly testing during the treatment process were nearly evenly split, with 20.7% agreeing and 18.3% rather agreeing, while 23.2% tended to disagree. Furthermore, 30.5% of respondents indicated that they rather disagreed with the necessity of pregnancy testing after the cessation of treatment.

More than half (63.4%) of the prescribers emphasized the importance of effective contraception and advised patients to contact their GP and/or gynecologist to discuss the possibility of using a contraceptive (Table 10).

More than half of the responding pharmacists always informed patients about the importance of effective contraception and recommended contacting a doctor if pregnancy was suspected (56.8% and 62.2%, respectively) (Table 11). In response to the open-ended question regarding what had specifically changed in their consultations (nine responses), pharmacists admitted that now they spent more time during consultations reminding patients about the teratogenic properties of the medication and emphasizing the risks associated with pregnancy.

## 4. Discussion

To understand the context of HCPs’ adherence to the PPP, it is essential that we recognize the barriers that they face in implementing these recommendations. Several studies have focused on identifying and examining the possible barriers that HCPs encounter when trying to follow various clinical guidelines in general. According to these studies, the barriers for HCPs can be thematically divided into three groups: knowledge-related barriers (a lack of general awareness or specific details of the guidelines), attitude-related barriers (such as a lack of outcome expectancy, inertia in previous practice, and a lack of agreement with guideline recommendations), and behavior-related barriers (patient factors, unclear or confusing recommendations, organizational constraints, and a lack of time) [17,18,19]. These findings could be relevant in understanding the reasons behind the insufficient implementation of the PPP, as the PPP requirements are often presented more as recommendations, with no consequences for HCPs in cases of omission.

With this context in mind, our study aimed to investigate the current knowledge, attitudes, and behaviors of prescribers and pharmacists regarding the teratogenicity of valproate and the effectiveness of PPP materials in Latvia.

### 4.1. Knowledge

In our study, both prescribers and pharmacists demonstrated an awareness of the teratogenic risks associated with valproates, as reflected in their questionnaire answers; however, the sources of information differed significantly. For prescribers, the main source of information was professional organizations, while for pharmacists, it was academic training. This may correlate with the median age of the respondents: the majority of pharmacists were students in 2018, whereas prescribers were typically middle-aged or older.

Regarding PPP measures, the most recognized by the prescribers were the discussion and signing of the RAF, which is a somewhat uncommon finding compared to results from other countries [9]. No objections to this PPP measure were reported by Latvian respondents; in contrast, prescribers in Denmark felt that the discussion and signing of the RAF could worsen their relationships with their patients [9]. For pharmacists, the most recognized PPP material was the warning symbol, which aligns with results from other EU countries, possibly due to the availability and ease of use of the material.

### 4.2. Attitudes

Prescribers were relatively enthusiastic about PPP materials and exhibited a desire to use all of them in the future. Materials such as the signing and discussion of the RAF received the highest scores. In contrast, the HCP guide and DHCP letter received the lowest scores, which corresponds with the findings observed in the EU results and is justified in the open-ended responses. The DHCP letter is viewed as a one-time notification that is not necessary for daily use; however, it is considered informative. As for the HCP guide, it is often absent from the workplace, which contributes to its lack of use.

Pharmacists also expressed a desire to use PPP materials in their work, with the warning symbol being the most valued material.

A significant number of prescribers believed that they were not responsible for providing information about the PPP for valproate-containing medications. This sentiment was more commonly expressed among GPs; however, other prescribers also mentioned it. Considering that in Latvia, GPs continue to prescribe valproates after the initial decisions made by neurologists or psychiatrists, it is crucial that we better involve GPs in the implementation of RMMs and emphasize the necessity of reviewing the PPP conditions each time that valproate is prescribed to women of reproductive age.

Pharmacists also tend to shift the responsibility of informing patients onto prescribers. They justify this by citing a lack of time for a thorough consultation in the community pharmacy environment, as well as the belief that it is not their role to provide such information, given that the prescriber is the one who makes the decision. If the medication has already been prescribed, they feel it would be inappropriate to comment on it. Altogether, this can lead to a situation where, for example, a GP relies on the initial prescriber to discuss the teratogenicity of valproate, while a pharmacist depends on the GP to remind the patient about the same issue. As a result, the patient remains unprotected from potential risks.

### 4.3. Behaviors

Overall, prescribers positively evaluate the impact of PPP materials on their prescribing and consultation practices, while pharmacists acknowledge that their behaviors have not changed since the introduction of the PPP. These opinions slightly contradict the findings in the knowledge section, where most pharmacists reported having become aware of the teratogenic risks of valproate in the last two years (e.g., around 2018). However, this can be explained by the fact that most pharmacists were educated through academic training, which suggests they may not have been influenced by the PPP materials themselves.

Regarding current practices, prescribers demonstrate selectiveness in prescribing valproates to women of reproductive age and, more generally, to patients overall. While there were no additional questions allowing prescribers to elaborate further on their prescribing strategies, it is possible that some doctors choose not to initiate treatment with valproate at all to avoid any risks and responsibilities related to patient care. This corresponds with another study on valproate prescription statistics in Latvia and may indicate a so-called “blanket” decision, when healthcare professionals (HCPs) are overly cautious and refrain from prescribing medication even when it could and should be the first choice [3]. This theme requires further exploration since, in some cases, valproate could be more effective than other medications, and it would not be appropriate to withhold it due to excessive caution. Regular education for HCPs and possibly Q&A sessions should be organized to prevent mistreatment.

The responses to questions regarding the performance of pregnancy tests before, during, and after treatment were generally low, particularly in comparison to the results from the EU. Given the significance of this aspect of the PPP and pharmacists’ belief that such information should originate from prescribers, enhancements in this area are essential. Nevertheless, the majority of prescribers emphasize the importance of effective contraception, which can help mitigate potential pregnancy-related risks. However, many prescribers appear to be inclined to refer questions regarding contraception to other specialists, as indicated by 59.8% of respondents who agree or rather agree with this practice, while none (0%) disagree. As a result, there is a lack of confidence in the topic of contraception being adequately addressed and discussed.

A similar situation is observed among pharmacists: while respondents actively emphasize the importance of contraception and recommend consulting a doctor if pregnancy is suspected, there is no consensus regarding the necessity of conducting pregnancy tests before or during treatment with valproates. A notable proportion of respondents (37.8%) indicated that treatment should be discontinued if pregnancy is suspected. Although pharmacists do not have the opportunity to elaborate on their specific recommendations in such cases, the decision to discontinue valproate therapy should be made only after a discussion with the prescriber, even if pregnancy is suspected.

### 4.4. Limitations

This study is limited by the relatively small sample size, which may affect the generalizability of the findings. While valuable insights were gathered from the healthcare professionals involved, a larger sample would enhance the robustness of the results and provide a more comprehensive understanding of their perspectives. Furthermore, the study questionnaires must be consistent across all eight countries to ensure that the results can be accurately generalized to the broader population. However, the questionnaire excluded individuals who were not pharmacists, such as pharmacy assistants, hospital pharmacists, and pharmacy students, because their status and work responsibilities may differ significantly across different EU countries. In Latvia, the responsibilities of pharmacy assistants in the field of dispensing medicines do not differ from those of pharmacists; therefore, understanding their experiences and habits would be important in reflecting the overall situation. Moreover, it would expand the sample size. The recruitment strategies for pharmacists (e.g., emails to pharmacies, the article on the Internet, and social networks) and electronic format of the survey could also be considered to be limiting factors. The majority of respondents in the survey were young specialists, and the median age of the sample was the lowest among all EU countries—31 years.

## 5. Conclusions

This study demonstrates that both prescribers and pharmacists in Latvia see their knowledge of the teratogenic risks associated with valproates and their familiarity with the aims and materials of the PPP as being satisfactory. However, the majority of HCPs believe that the valproate PPP requires amendments, as not all materials and measures can be used efficiently in practice.

The roles and responsibilities of each category of HCP need to be evaluated and clearly stated, as both prescribers and pharmacists exhibit habits of referring patients to other specialists and omitting detailed discussions. Further research is necessary to evaluate valproate prescription strategies and prescribers’ attitudes, as well as to explore HCPs’ perspectives on this subject in greater detail. It would be beneficial to re-evaluate the PPP materials at a general level and make them more convenient and accessible for all HCP groups.

## Figures and Tables

**Table 1:** Content of the Pregnancy Prevention Program for HCPs in Latvia.

Educational Material	Prescribers	Pharmacists
Healthcare professional (HCP) guide	X	X
DHCP letter	X	X
Patient reminder card	X	X
Review the risk acknowledgment form	X	
Sign the risk acknowledgment form	X	
Patient guide	X	
Warning symbol on outer packaging		X
Pharmacist checklist		X

**Table 2:** Characteristics of the respondents (prescribers—N = 102, pharmacists—N = 48).

Characteristics	Variables	Prescribers N (%)	Pharmacists N (%)
Age	Median (SD) age	52	31
	Range of age	25–82	22–62
Gender	Male	23 (22.5%)	4 (8.3%)
	Female	78 (76.4%)	43 (89.6%)
	No gender statement	2 (2.0%)	1 (2.1%)
Work experience	0–5 years	13 (12.7%)	23 (47.9%)
	6–10 years	8 (7.8%)	13 (27.1%)
	11–20 years	17 (16.7%)	6 (12.5%)
	21–30 years	31 (30.4%)	4 (8.3%)
	>31 years	34 (33.3%)	2 (4.2%)
Specialization	GP	36 (35.3%)	-
	Neurologist	24 (23.5%)	-
	Psychiatrist	31 (30.4%)	-
	Other	12 (11.8%)	-

**Table 3:** Knowledge of prescribers and pharmacists about the teratogenicity of valproate.

	Prescribers N (%)	Pharmacists N (%)
Time that they have been familiar with the PPP		
<2 years	21 (20.6)	23 (47.9)
2–5 years	20 (19.6)	16 (33.3)
>5 years	57 (55.9)	9 (18.8)
Information resources		
Health authorities	39 (39.8)	22 (45.8)
State Medicines Agency	41 (41.8)	16 (33.3)
Professional organization	68 (69.4)	6 (12.5)
Colleagues	24 (24.5)	17 (35.4)
Professional journals	45 (45.9)	11 (22.9)
Pharmaceutical companies (printed/electronical materials)	31 (31.6)	10 (20.8)
Internet	15 (15.3)	10 (20.8)
Symposia or conferences	40 (40.8)	1 (2.08)
Academical training	35 (35.7)	24 (50.0)
During post-academic training/continuous professional education	42 (42.9)	5 (10.4)

**Table 4:** The use of PPP materials by prescribers (N = 95).

	I Have Seen It Before But I Don’t Use It	I Used or Am Using It	No, I Have Not Seen It	I Am Not Sure
	N (%)			
Healthcare professional guide	15 (15.8)	30 (31.6)	40 (42.1)	10 (10.5)
Patient guide	18 (18.9)	38 (40.0)	32 (33.7)	7 (7.4)
RAF discussion	7 (7.4)	52 (54.7)	29 (30.5)	9 (9.5)
RAF signing	8 (8.4)	51 (53.7)	30 (31.6)	6 (6.3)
Patient reminder card	13 (13.7)	49 (51.6)	22 (23.2)	11 (11.6)
DHPC	33 (34.7)	28 (29.5)	23 (24.2)	14 (14.7)

**Table 5:** The use of PPP materials by pharmacists (N = 44).

	I Have Seen It Before But I Don’t Use It	I Used or Am Using It	No, I Have Not Seen It	I Am Not Sure
	N (%)			
Healthcare professional guide	8 (16.7)	7 (14.6)	26 (54.2)	3 (6.3)
Pharmacist checklist	5 (10.4)	15 (31.3)	21 (43.8)	3 (6.3)
Warning symbol on the outer carton	6 (12.5)	34 (70.8)	1 (2.1)	3 (6.3)
Patient reminder card	5 (10.4)	7 (14.6)	21 (43.8)	11 (22.9)
DHCP letter	22 (45.8)	14 (29.2)	5 (10.4)	3 (6.3)

**Table 6:** Attitudes of prescribers toward the use of PPP materials in the future.

	HCP Guide	Patient Guide	RAF Discussion	RAF Signing	Patient Reminder Card	DHCP Letter
	N = 65	N = 57	N = 45	N = 44	N = 46	N = 67
N (%)						
Very likely	4 (6.2)	9 (15.8)	20 (44.4)	11 (25.0)	8 (17.4)	12 (17.9)
Likely	24 (36.9)	29 (50.9)	13 (28.9)	15 (34.1)	19 (41.3)	29 (43.3)
Neither unlikely nor likely	18 (27.7)	13 (22.8)	12 (26.7)	15 (34.1)	13 (28.3)	16 (23.9)
Unlikely	13 (20.0)	6 (10.5)	0 (0.0)	3 (6.8)	1 (2.2)	8 (11.9)
Very unlikely	6 (9.2)	0 (0.0)	0 (0.0)	0 (0.0)	5 (10.9)	2 (3.0)

**Table 7:** Attitudes of pharmacists toward the use of PPP materials in the future.

	Healthcare Professional Guide	Pharmacist Checklist	Warning Symbol	Patient Reminder Card	DHCP Letter
	N = 37	N = 28	N = 9	N = 36	N = 30
N (%)					
Very likely	4 (10.8)	3 (10.7)	3 (33.3)	5 (13.9)	10 (33.3)
Likely	20 (54.1)	11 (39.3)	6 (66.7)	12 (33.3)	10 (33.3)
Neither unlikely nor likely	2 (5.4)	3 (10.7)	0 (0.0)	9 (25.0)	3 (10.0)
Unlikely	6 (16.2)	8 (28.6)	0 (0.0)	6 (16.7)	7 (23.3)
Very unlikely	5 (13.5)	3 (10.7)	0 (0.0)	4 (11.1)	0 (0.0)

**Table 8:** Changes in prescribing/dispensing practice since the introduction of the PPP in 2018.

	Prescribers (N = 91)	Pharmacists (N = 38)
Did your behavior change after 2018	N (%)	N (%)
Certainly yes	25 (27.4)	5 (13.2)
Probably yes	26 (28.6)	6 (15.7)
Not sure	16 (17.6)	7 (18.4)
Probably no	15 (16.5)	18 (47.3)
Certainly no	9 (9.9)	2 (5.3)

**Table 9:** Prescription practices.

	Agree	Rather Agree	Rather Disagree	Disagree
Prescription practices	N (%)			
No prescription at all	24 (29.3)	13 (15.9)	11 (13.4)	22 (26.8)
No prescription to women of reproductive age	39 (47.6)	21 (25.6)	15 (18.3)	5 (6.1)
Selective with prescription to women of reproductive age	58 (70.7)	10 (12.2)	1 (1.2)	1 (1.2)
Discontinuing prescription for women who plan or suspect pregnancy	49 (59.8)	8 (9.8)	12 (14.6)	3 (3.7)
Referral to a medical specialist when a pregnancy is suspected	63 (76.8)	10 (12.2)	0 (0.0)	0 (0.0)

**Table 10:** Counseling on contraception.

	Agree	Rather Agree	Rather Disagree	Disagree
	N (%)			
Inform patients about the importance of effective contraception	52 (63.4)	8 (9.8)	1 (1.2)	1 (1.2)
Prescribe effective contraception	15 (18.3)	11 (13.4)	7 (8.5)	5 (6.1)
Advise patients to contact their general practitioner/gynecologist to discuss effective contraception	49 (59.8)	13 (15.9)	0 (0.0)	0 (0.0)

**Table 11:** Information provision habits among pharmacists.

	Always	Often	Seldom	Never
	N (%)			
Inform/remind patients about the use of effective contraception	21 (56.8)	8 (21.6)	5 (13.5)	3 (8.1)
Recommend stopping treatment if pregnancy is suspected	14 (37.8)	6 (16.2)	7 (18.9)	10 (27.0)
Recommend contacting their doctor if pregnancy is suspected	23 (62.2)	7 (18.9)	4 (10.8)	3 (8.1)
Emphasize the need for pregnancy tests before/during treatment	9 (24.3)	11 (29.7)	8 (21.6)	9 (24.3)

## Data Availability

Upon reasonable request, study authors will provide access to data.
